# Measuring Chinese English-as-a-foreign-language learners’ resilience: Development and validation of the foreign language learning resilience scale

**DOI:** 10.3389/fpsyg.2022.1046340

**Published:** 2022-11-28

**Authors:** Nianyu Guo, Rui Li

**Affiliations:** School of Foreign Languages, Hunan University, Changsha, China

**Keywords:** foreign language learning resilience scale, English as a foreign language, ego resilience, metacognitive resilience, social resilience

## Abstract

Despite the growing body of research on the factors of resilience in diverse fields, there is still a dearth of particular attention on foreign language learning resilience. To fill the gap, this study seeks to develop the foreign language learning resilience scale (*FLLRS*) to measure its psychometric scale reliability and validity in Chinese English-as-a-foreign-language contexts. Valid data were collected from 313 Chinese English-as-a-foreign-language college students who voluntarily participated in the survey. The *FLLRS* was validated based on a series of reliability (e.g., item analysis, split-half reliability and internal consistency) and validity (e.g., construct validity, convergent validity and discriminant validity) tests. Results suggested that the 19-item *FLLRS* presented three factors: *ego resilience*, *metacognitive resilience* and *social resilience*. Besides, all the three factors contributed high effects to foreign language learning resilience. Among the three factors, *metacognitive resilience* was found to have the highest path coefficient, followed by *social resilience*, with *ego resilience* having the lowest. The validated scale could advance knowledge in the field of second language acquisition regarding how learners’ individual differences, emotional factors and the contextual antecedents may affect foreign language learning resilience.

## Introduction

Resilience is about individuals’ capability of making positive adaptation to the stressful and challenging situations (Lazarus and Folkman, 1984; Ungar, 2008; Mansfield et al., 2016). Individuals may encounter a plethora of difficulties brought by high-demanding assignments, negative relationship with peers and teachers and the imposition coming from their families (Yun et al., 2018; Brewer et al., 2019; Trigueros et al., 2020). Resilient individuals are more capable of dealing with these adversities (Axford et al., 2014; Broadbent and Poon, 2015; Chan et al., 2021), while others are less inclined to absenteeism (Seçer and Ulas, 2020), psychological disorders (Zhang et al., 2020), and even self-handicap (Hunsu et al., 2021).

In the past decade, an increasing number of studies have been designed to validate measures for resilience in clinical psychology (Wagnild and Young, 1993; Connor and Davidson, 2003), education psychology (Block and Kreman, 1996; Cassidy, 2016), mathematics (Martin and Marsh, 2008a, 2008b), engineering learning (Hunsu et al., 2021) and other contexts (van der Meer et al., 2018), which might provide some valuable insights into understanding the factors of resilience. Nevertheless, very few studies to date have sought to dig deeper into the phenomenon of resilience among students in foreign language (FL) contexts (Nguyen et al., 2015; Yun et al., 2018; Sudina and Plonsky, 2021), as it is crucially important to facilitate FL learning process when they can make positive adaptation to such adversities as FL anxieties (Li, 2022a,b), FL guilt and shame (Teimouri, 2018), and untimely feedback from teachers due to high teacher-student ratio (Li, 2021). To our knowledge, most of these studies aimed to explore predictors or correlates of resilience among FL learners in South Korea (Kim et al., 2018, 2019; Kim and Kim, 2021), Western Canada (Lou and Noels, 2020a,b) and Southwestern America (Sudina and Plonsky, 2021) without a detailed scrutiny of its factors. Thus, it is necessary to develop and validate a FL resilience scale by expanding the dimensions under analysis.

To this end, this study measures resilience among Chinese English as a FL (EFL) learners, so large a population that should not be ignored (Li, 2021), and validates a Chinese version of the foreign language learning resilience scale (*FLLRS*) to provide a more up-to-date vision on this issue. In doing so, drawing on existing studies (e.g., Zhang et al., 2021), the instrument scale development should be validated by both the explanatory factor analysis (EFA) and confirmatory factor analysis (CFA) methods. More specifically, while the EFA lacks the goodness-of-fit indexes that CFA offers, it considers the cross-loadings of all items and avoids zero cross-loadings of the related construct, which enables us to gain a clear understanding of the possible dimensions. On the other hand, while the CFA ignores cross-loadings of the measurement model, it can not only provide sufficient goodness-of-fit indexes, but also further test the predictive power of the constructs. To this end, it aims to (a) understand the factors underlying the *FLLRS*; while (b) further validating the scale with CFA to understand the different effects of the factors in Chinese EFL contexts.

## Literature review

### Theoretical framework of resilience

In regard to resilience, this study adopts Lazarus and Folkman (1984) stress and coping theory (SCT) as the framework, as it highlights that resilience tends to occur when “judgment of person-human relationship is stressful hinges on personal, cognitive and situational appraisals” (Lazarus and Folkman, 1984, p. 21). Motivated by the SCT, three components – personal, cognitive and situational appraisals – are elaborated in the remainder of this section, respectively.

Personal appraisal is related with *commitments* and *beliefs*. *Commitments* refer to what is important or meaningful to an individual. For instance, when resilient EFL learners perceive English learning as an important activity, they will appraise or evaluate it as something meaningful and “maintain valued ideals to achieve desired goals” (*ibid.*, p.56). *Beliefs* are defined as “personally formed configurations” (*ibid.*, p.65) that usually operate at a tacit level to determine the understanding of a fact. For instance, perseverant EFL learners might hold the belief that English cannot be mastered for a short period of time.

Cognitive appraisal “can be readily understood as the process of categorizing an encounter, and its various facets, with respect to its significance for well-being” (*ibid.*, p.31). Lazarus and Folkman (1984) further classified it into *primary appraisal* (“Am I in trouble or being benefited, now or in the future, and in what way?”) and *secondary appraisal* (“What if anything can be done about it?”). The *primary appraisal* refers to the process in which an individual evaluates the relationship with the situation he/she locates. The *secondary appraisal*, on the other hand, is about an individual’s capacities to deal with the stressful and challenging situations. During the process, resilient individuals may adopt a series of cognitive and metacognitive resources, such as perceiving, monitoring, judging and discriminating information, to deliberately focus on the positive aspects of what is happening or solving the underlying distress with positive emotions (Li et al., 2021).

Situational appraisal is defined as the identification of situational properties that “may potentially be harmful, dangerous and threatening” (Lazarus and Folkman, 1984, p. 82). In other words, resilient individuals are able to make the positive adaption to the stressful situations by taking advantage of the limited resources. For instance, resilient EFL learners who have fully evaluated the situational properties would seek help from classmates or teachers in times of English learning difficulties.

Taken together, it should be noted here that, despite the three appraisals being separately classified, they are closely interconnected to shape the capability of resilience. The SCT contributes to advancing our understanding of personal, cognitive and situational appraisals with respect to the domain-general resilience, and paves the way for understanding the factors of FL learning resilience in particular.

### Types of resilience

Drawing on the theoretical insights of SCT, the understanding of resilience’s factors has long been a focus of interest in relation to personal, cognitive and situational factors. Accordingly, the most typical types of resilience are *ego resilience* (Block and Kreman, 1996; Chen et al., 2020; Chen and Padilla, 2022), *cognitive resilience* (Cassidy, 2016; Ang et al., 2021; Jahedizadeh et al., 2021) and *social resilience* (Ungar, 2008; Ungar et al., 2021), among others.

#### Ego resilience

*Ego resilience* refers to how individuals respond to adversities they are experiencing, and consider their capacity to recover from the adversities (Maltby et al., 2015). In the social psychology contexts, researchers (Block and Kreman, 1996) have explored the constructs of *ego resilience* and proposed one of the most influential scales – 14-item *Ego-Resiliency Scale (ER89)* – having two factors: *openness to life experience* and *optimal regulation*. Wagnild and Young (1993) also explored the factors of *ego resilience* from a 25-item scale and obtained a two-factor structure, *viz. personal competence* and *acceptance of self and life*. Similarly, Connor and Davidson (2003) developed a 25-item *Connor–Davidson Resilience Scale (CD–RISC)* in a clinical context, and extracted five factors – Factor 1 (*personal competence, high standards and tenacity*), Factor 2 (*trust in one’s instincts, tolerance of negative effect, strengthening effects of stress*), Factor 3 (*positive acceptance of change, and secure relationships*), Factor 4 (*control*), and Factor 5 (*spiritual influence*).

#### Cognitive resilience

*Cognitive resilience* refers to individuals who seek some positive cognitive strategies (e.g., goal setting, goal planning, help-seeking and control) and activate their cognitive mechanisms (e.g., growth mindset, inhibition control, working memory, cognitive flexibility and cognitive emotion regulation) to deal with the challenging and stressful situations (Cassidy, 2016; Lou and Noels, 2020a,b). In the educational contexts, for instance, Cassidy (2016) developed an *Academic Resilience Scale (ARS-30)* to measure learners’ cognitive, strategic and successful adaptation to academic challenges. The *ARS-30* has three factors, namely Factor 1 (*perseverance*), Factor 2 (*reflecting and adaptive help-seeking*), and Factor 3 (*negative affect and emotional response*). In a recent attempt, van der Meer et al. (2018) developed a two-factor *Resilience Evaluation Scale (RES)* – *self-confidence* and *self-efficacy* – to understand how individuals handle academic difficulties in stressful and challenging situations.

#### Social resilience

*Social resilience* is the process of “navigating the necessary resources” by positively connecting with parents, schools and communities (Ungar, 2019, p.2). In psychiatric contexts, Friborg et al. (2003) developed a five-factor *Resilience Scale for Adults (RSA)* – *personal competence*, *social competence*, *family coherence*, *social support* and *personal structure* – to investigate how intrapersonal and interpersonal factors help individuals make positive adaptation to stressful situations. Using mixed methods, Liebenberg et al. (2011) validated a three-factor *Child and Youth Resilience Measure (CYRM-28)* in a cross-cultural context – *individual factors*, *caregiving or relational factors*, and *contextual components*. Insights gained from the *CYRM-28*, the exploration of *social resilience* has been conducted in other equally important contexts, such as psychotherapeutics (Chan et al., 2021), education psychology (Lavy and Ayuob, 2019), and social psychology (Ungar et al., 2021).

While the aforementioned studies provide valuable insights into the types of resilience, there are some limitations of the existing literature. On the one hand, it is evident to note that most of these studies examine only one type of resilience but fail to integrate *ego resilience*, *cognitive resilience* and *social resilience* in a study, and “most effectively differentiate the factors that are (and are not) components, causes, and correlates” (Martin and Marsh, 2009, p. 353). On the other hand, while three types of resilience driven by the SCT in the domain-general contexts have been thoroughly investigated, understanding its factors may be context-specific and cannot be easily generalized to EFL learning contexts. In other words, it remains largely unknown whether these studies could be extended to FL learning and there is still substantial room for further research on resilience in FL learning contexts.

### Resilience in FL learning contexts

In FL learning contexts, resilience is defined as the ability to “overcome stress and maintain high mental stamina” in FL learning adversities (Kim et al., 2018, p. 56). To date, an emergent body of research (Nguyen et al., 2015; Kim et al., 2019; Lou and Noels, 2020a,b; Sudina and Plonsky, 2021; Chen and Padilla, 2022) seeks to examine the relationship between resilience and other variables in the FL learning contexts, including stress and coping (Gregersen et al., 2021), (de-)motivation and language proficiency (Nguyen et al., 2015; Kim et al., 2018, 2019; Kim and Kim, 2021), emotions, creativity, or growth mindsets (Sudina and Plonsky, 2021; Chen and Padilla, 2022), and buoyancy, grit and academic perseverance (Lou and Noels, 2020a,b). For instance, Kim et al. (2018) collected qualitative data from 23 EFL learners and nine teachers, and identified four components of FL resilience: *social support*, *emotional regulation*, *a clear learning goal* and *tenacity in EFL learning*. Shortly afterwards, they (Kim et al., 2019) further collected quantitative data from 367 South Korean elementary school students to explore the impact of FL resilience on (de) motivation and language proficiency. Results indicated that FL resilience consists of *metacognitive adaptation*, *sociability*, *optimism*, *perseverance* and *communicative efficacy*, and it was reported to have a direct impact on motivation. In a recent study, informed by academic resilience (Martin and Marsh, 2008a,b; Sudina and Plonsky, 2021) collected data from 360 Fl learners based on an 8-item *Foreign Language Buoyancy Scale (FLBS)* to investigate the related correlates of FL learning perseverance. Using EFA, they obtained two components of FL resilience: *coping with poor grades and criticism* and *dealing with study stress*.

### The present study

What emerges from the above review is that despite the growing diversity of studies providing some valuable insights into understanding the correlates of FL resilience, research gaps on its factors remain open for debate. First, on a scrutiny of the studies involved, some of these lack a solid theoretical framework for FL resilience. For instance, as the first scale measuring FL resilience, eight items of *FLBS* that were directly adopted based on the domain-general academic resilience (Martin and Marsh, 2008a, 2008b) may discount the context-specificity and theoretical foundations. Similarly, Kim et al. (2018, 2019) preliminary attempts for FL resilience also failed to consider the theoretical underpinnings of FL learning resilience. Second, given the increased scrutiny for relationship between resilience and other variables in FL learning contexts, there is a desperate lack of research that should validate and measure the factors of FL resilience. Third and importantly, while FL resilience of the existing studies was measured for very specific population, namely primary, secondary school, and college-level students in South Korea (Kim et al., 2018, 2019; Kim and Kim, 2021), college students in Western Canada (Lou and Noels, 2020a,b) and Southwestern America (Sudina and Plonsky, 2021), its applicability to Chinese EFL learners remains largely underexplored. As Sudina and Plonsky (2021, p.13) put it, “future studies need to cross-validate the factors with a new sample of FL learners.”

To fill a void in this line, the purposes of the study are two-fold: Motivated by the theoretical framework of SCT, the first aim is to develop and validate the *FLLRS*, so as to profile the factors underlying the *FLLRS* in Chinese EFL contexts. A second aim is to understand the extent to which different factors may contribute to the overall FL resilience. Consequently, two research questions are to be addressed as follows.

Research question 1 (RQ1): What are the factors underlying the *FLLRS* in Chinese EFL contexts?

Research question 2 (RQ2): How do the factors contribute to FL learning resilience?

## Materials and methods

### Participants

A total of 420 EFL undergraduate students recruited from four Double First-Class (*viz.* world class universities and disciplines) universities in central China volunteered and consented to participate in the online survey^1^ through the convenient sampling method in the classroom. It takes roughly 30 min for the participants to complete the questionnaire. The study was approved by the Ethics Committee of Hunan University. Students of the Double First-Class universities were chosen for the following considerations. On the one hand, these four prestigious universities evenly distributed across similar levels of higher education institutions in central China, warranting the homogeneity of the data collected. On the other hand, the emphasis in the Double First-Class universities on an international outlook in general, and on a quality education in English in particular, enables students to acquire a good mastery of English. Thus, students of these universities are more likely to achieve a high level of resilience if they encounter difficulties with the English language. There was neither incentive for completing the survey, nor was there any penalty for not completing the questionnaire. The data of 107 students were removed due to their failure in trap questions, resulting in 313 valid data for analysis. Among the 313 participants, only those in Year 1 (93.6%, *N* = 293) and Year 2 (6.4%, *N* = 20) were investigated because non-English major students of Year 3 and 4 did not attend English class in China. There were 119 males (aged: 18.43 ± 1.17) and 195 females (aged: 18.40 ± 0.72). The average ages of the participants were 18.41 (*SD* = 0.91) years old. These EFL learners are of intermediate proficiency level based on their national-scale college English entrance test scores of 117.09 ± 16.83 (full score: 150). According to Boateng et al. (2018), the minimum number of participants needed for the analysis to be valid should follow the rule of thumb, which requires at least 10 respondents for each scale item. As such, the total of 313 valid participants which was higher than 240 (24 × 10) met the criteria.

### Item generation procedures

Before initial item generation, item development for the *FLLRS* was based on the theoretical framework of SCT (Lazarus and Folkman, 1984), and the synthesis of related existing studies regarding resilience research in educational psychology (Wagnild and Young, 1993; Block and Kreman, 1996; Connor and Davidson, 2003; Friborg et al., 2003; Campbell-Sills and Stein, 2007; Cassidy, 2016; van der Meer et al., 2018; Chen et al., 2020; Ungar et al., 2021), and insights provided by skilled researchers and learners of similar background. We generated an initial pool of 24 items for three proposed factors: *ego resilience* (8 items, e.g., “I am curious about the new knowledge when I study a FL.”), *metacognitive resilience* (8 items, e.g., “I would use the feedback to improve my FL.”) and *social resilience* (8 items, “When I am encountered with difficulties in FL learning, I would seek help from my teachers.”).

After the initial item generation, detailed questionnaire development procedures were observed as follows. First, questionnaire items of the *FLLRS* were first translated into simplified Chinese. Second, Chinese version of the items was translated back to English by a teacher of English translation using a forward-backward translation (Li, 2021; Li et al., 2021). The high similarity between two versions confirmed its accuracy. Minor adjustments to wording and formatting were made accordingly. Third, face validity of the items was reviewed and confirmed by another five researchers, including two researchers in educational psychology and three in second language acquisition. Fourth, to ensure that the questionnaire items caused no misinterpretations and were fully understood, wording of the items was reviewed and discussed in a pilot study of 32 EFL learners with similar educational background. Minor adjustments to wording were further resolved by consensus through discussions. The initial 24 items (Appendix 1) had a 7-point Likert scale survey anchored on “1 = strongly disagree” and “7 = strongly agree.”

### Data analysis

A series of explanatory and confirmatory factor analyses was performed in an attempt to solve the two research questions. To gain a better understanding of the factors (RQ1), results of psychometric validity and reliability of the *FLLRS* were reported first. In doing so, item analysis, reliability analyses (internal consistency and split-half reliability) and EFA were conducted. For item analysis, statistical comparison of 27% upper and lower items should be made to ensure the discrimination of each item. For reliability analyses, the cut-off values of Cronbach’s α and split-half reliability should be over 0.70 (Li et al., 2019). Second, to answer RQ2 regarding the contribution of the factors, CFA of the *FLLRS* reporting the measurement and structural model was carried out accordingly.

## Results

In what follows, results corresponding to research questions were presented in the remainder of this section.

### Psychometric validity and reliability of the FLLRS

#### Item analysis

Item analysis was performed with independent samples *t*-test to compare the statistical difference of responses between 27% upper items (*viz.* the highest 27% ratings) and 27% lower items (*viz* the lowest 27% ratings) based on participants’ rating scores of the 7-point Likert scale (Li, 2021). The results indicated that significant between-group difference was obtained for each of the 24 items (all *p*s < 0.001), suggesting the high discrimination of each item appropriate for further analysis.

#### EFA

Drawing on Kaiser (1970), the EFA was adopted with a principal components analysis (PCA) and Varimax rotation (e.g., Dewaele and MacIntyre, 2016) to determine which of 24 items clustered together to form general factors. Those factors that had more than one item with an eigenvalue ≥1.00 and the factor loadings greater than 0.4 on the intended factor but less than 0.4 on any other factor were retained.

Bartlett’s test of sphericity (*χ*^2^ = 5088.039, *df* = 276, *p* = 0.000) and the Kaiser-Meyer-Olkin measure of sampling adequacy (KMO = 0.926) exceeded the recommended value of 0.6 (Kaiser, 1970). The first factor analysis yielded five factors, which accounted for 68.918% of the total variance. However, the cross-loading problems suggested further iterative deletion and analysis. After the iterative deletion of five cross-loading items (Item 4, 5, 7, 8 and 16, see Appendix 1 for more), factor analysis of the remaining 19 items that did not have the cross-loading problems met the criteria with satisfied Bartlett’s test of sphericity (*χ*^2^ = 4077.672, *df* = 171, *p* = 0.000) and the KMO of 0.925. Table 1 demonstrated the results of EFA regarding factors, items, item means and standard deviations, Cronbach’s α and factor loadings, respectively.

**Table 1:** Results of explanatory factor analysis: Varimax rotated factor loadings.

Factor	Item	*M* ± *SD*	Cronbach’s *α*	Factor loadings		
				Factor 1	Factor 2	Factor 3
ER	–	5.332 ± 1.083	0.806	–	–	–
	1	4.86 ± 1.499		0.804		
	2	4.57 ± 1.479		0.756		
	3	5.60 ± 1.386		0.734		
	6	6.29 ± 1.030		0.544		
MR	–	4.861 ± 1.234	0.931	–	–	–
	9	4.92 ± 1.475			0.781	
	10	4.71 ± 1.446			0.722	
	11	4.80 ± 1.479			0.806	
	12	5.04 ± 1.489			0.717	
	13	4.98 ± 1.428			0.779	
	14	4.73 ± 1.480			0.786	
	15	4.84 ± 1.483			0.654	
SR	–	4.748 ± 1.177	0.900	–	–	–
	17	4.73 ± 1.445				0.735
	18	4.42 ± 1.487				0.741
	19	4.82 ± 1.554				0.687
	20	5.07 ± 1.458				0.715
	21	4.87 ± 1.460				0.715
	22	4.84 ± 1.546				0.624
	23	4.64 ± 1.732				0.596
	24	4.58 ± 1.563				0.744

M, mean; SD, standard deviation; factor loadings more than 0.50 are presented; ER, ego resilience; MR, metacognitive resilience; SR, social resilience; factor loadings lower than 0.500 were not presented.

In Table 1, three factors explained 64.986% of the total variance with robust factor loadings (>0.50) on the intended factor. Factor 1 (eigenvalue = 1.184) was labeled *ego resilience* (four items) and explained 14.936% of the variance, which refers to EFL learners’ personal attributes, such as perseverance, curiosity and energy, to recover from the FL learning adversities. Factor 2 (eigenvalue = 9.539) was labeled *metacognitive resilience* (seven items) and explained 25.601% of the variance, which means EFL learners may seek for metacognitive strategies, such as goal setting, goal planning, help-seeking and control, to deal with FL learning difficulties. Factor 3 (eigenvalue = 1.624) was labeled *social resilience* (four items) and explained 24.499% of the variance, which means EFL learners may establish positive social connection with parents, schools and communities to solve FL learning problems. The means (*M*) and standard deviations (*SD*) of each factor were presented in Table 1. All scored above 4, with *ego resilience* (5.332 ± 1.083) being the highest, followed by *metacognitive resilience* (4.861 ± 1.234), and *social resilience* (4.748 ± 1.177).

#### Reliability

##### Internal consistency

Internal consistency was assessed using Cronbach’s *α* for each structure (Table 2), which showed high reliability results as reflected in the Cronbach’s α for the overall scale (*α* = 0.940), *ego resilience* (*α* = 0.806), *metacognitive resilience* (*α* = 0.931) and *social resilience* (*α* = 0.900), respectively.

**Table 2:** Overall reliability and validity analysis of the measurement model.

	Reliability	Convergent validity	Discriminant validity		
			Latent variable correlations		
Factor	CR	AVE	ER	MR	SR
ER	0.810	0.542	**0.736**		
MR	0.930	0.656	0.651^**^	**0.807**	
SR	0.899	0.528	0.575^**^	0.689^**^	**0.727**

ER, ego resilience; MR, metacognitive resilience; SR, social resilience; CR, composite reliability; AVE, average variance extracted. *p < 0.05, **p < 0.01. Square roots of AVEs are shown as diagonal elements in bold type.

##### Split-half reliability

Split-half reliability was used to evaluate the internal reliability of the *FLLRS*. The *rhh* correlation between the two halves (First half: Item 1, 2, 3, 6, 9, 10, 11, 12, 13 and 14; Second half: Item 14, 15, 17, 18, 19, 20, 21, 22, 23 and 24) was 0.745, and the Spearman-Brown *rtt* for the overall scale was 0.854, indicating the high internal reliability of the scale for further analysis.

### CFA of the FLLRS

The confirmation of factors obtained from the EFA was further tested with CFA, *viz.* a technique used to understand the extent to which each factor affects the overall *FLLRS*.

#### Measurement model

The reliability, convergent validity and discriminant validity were reported in Table 2. The reliability of the measurement model was confirmed, as composite reliability was over the minimum of 0.60 (Hair et al., 2006). The validity of the measurement model was also confirmed, since values of average variance extracted (AVE) for each factor exceeded the threshold value of 0.05 and discriminant validity was higher than the corresponding latent variable correlations (Fornell and Larcker, 1981). As such, overall results of reliability and validity of the measurement model were confirmed, since all the values met the required criteria.

#### Structural model

Using the maximum likelihood method, the structural model was evaluated with six indices involved: normed chi-square, goodness-of-fit index (GFI), adjusted goodness-of-fit index (AGFI), comparative fit index (CFI), Tucker-Lewis index (TLI), and root-mean-square error of approximation (RMSEA), respectively. Results of these indices that were summarized in Table 3 met the suggested values (Li et al., 2021), indicating the appropriateness of the structural model.

**Table 3:** Model fit indices of confirmatory factor analysis.

Model fit indices	χ^2^/*df*	GFI	AGFI	CFI	TLI	NFI	RMSEA
Result	2.532	0.897	0.862	0.946	0.935	0.914	0.070
Suggested	<3	>0.90	>0.80	>0.90	>0.90	>0.90	<0.10
Evaluated	Good	Close	Good	Good	Good	Good	Good

GFI, goodness-of-fit index; AGFI, adjusted goodness-of-fit index; CFI, comparative fit index; TLI, Tucker-Lewis index; NFI, normed fit index; RMSEA, root-mean-square error of approximation.

**Figure 1:**
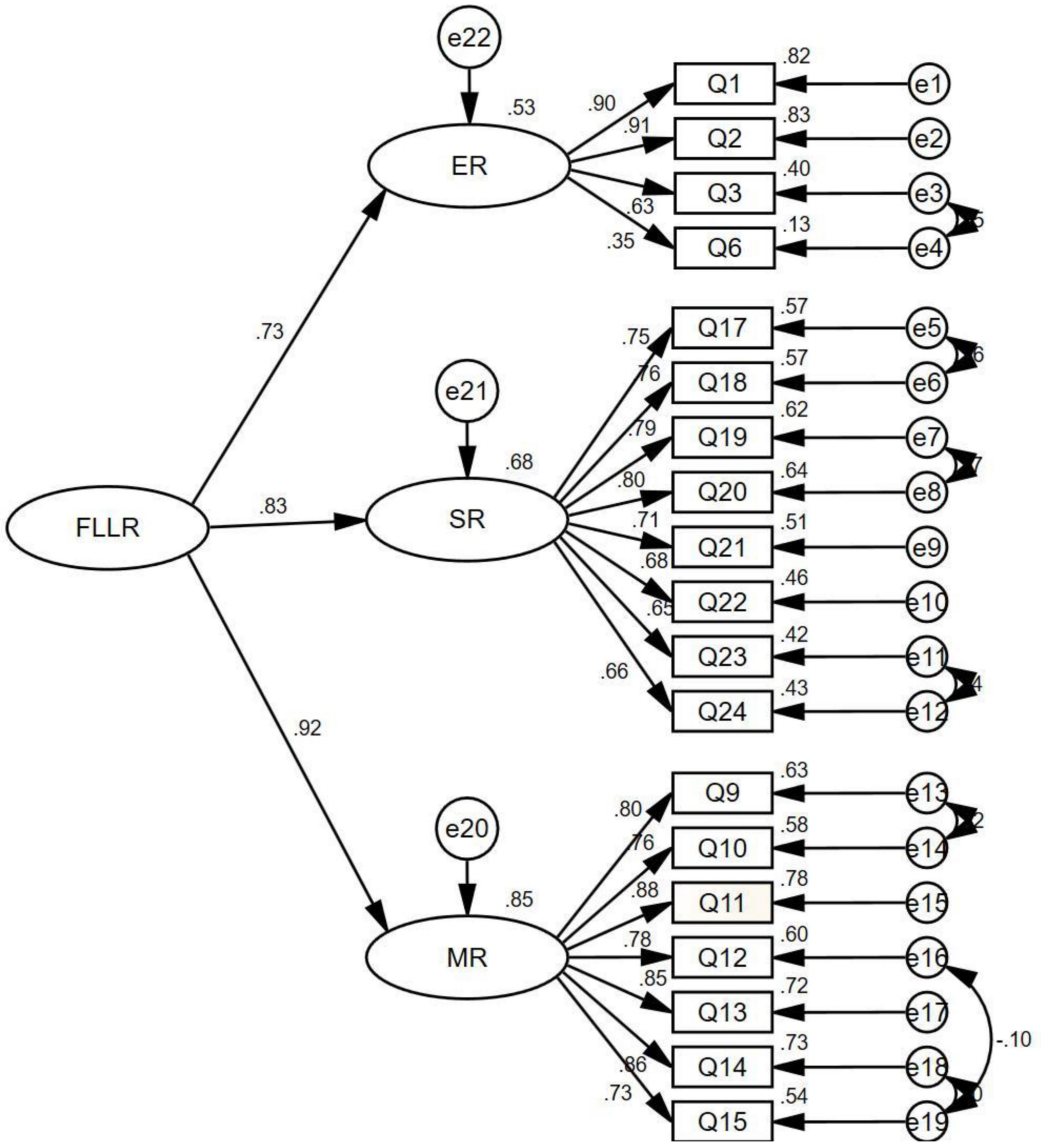
Confirmatory factor analysis results for the three-factor structure. *N* = 313, *χ^2^* = 359.597, *df* = 142, *p* < 0.001. FLLR, foreign language learning resilience; ER, ego resilience; SR, social resilience; MR, metacognitive resilience.

The structural model of *FLLRS* was validated and presented in Figure 1. It was found that all the three factors can positively predict EFL learners’ FL learning resilience with large effect sizes (0.25, 0.40, and 0.60 for small, moderate, and large, see Plonsky and Oswald, 2014), while the path coefficient of *metacognitive resilience* (*β* = 0.92, *p* < 0.001) was higher than that of *social resilience* (*β* = 0.83, *p* < 0.001) and *ego resilience* (*β* = 0.73, *p* < 0.001). Among the three factors, *ego resilience* has the lowest coefficient.

## Discussion

This study contributes to the field of second language acquisition (SLA) and adds to the emerging body of SLA literature by constructing and validating the factors of *FLLRS*. The deeper understanding of a new cognitive and conative factor in FL learning and teaching—FL learning resilience—is pedagogically crucial to the stakeholders (e.g., teachers, policy-makers, institutional leaders, etc.) who should pay particular attention to FL learners’ resilience in times of difficulties or adversities. Considering an increased focus on the language-specific correlates of perseverance in FL learning in general (Sudina and Plonsky, 2021), and FL resilience in particular (Nguyen et al., 2015; Kim et al., 2018, 2019; Lou and Noels, 2020a,b), the current study, drawing on the theoretical framework of SCT (Lazarus and Folkman, 1984), provides an FL-specific scale measuring EFL learners’ resilience, and explores the different impacts of factors on FL resilience. More specifically, this study first examined the factors of *FLLRS* with a series of reliability (e.g., item analysis, split-half reliability, and internal consistency) and validity (e.g., construct validity, convergent validity and discriminant validity) tests, which showed that the 19-item FLLRS has satisfactory psychometrical properties to be used as a validated scale in the FL learning contexts for future research. Additionally, the 19-item *FLLRS* was further validated from the CFA. Among the three factors, *metacognitive resilience* was found to have the highest path coefficient, followed by *social resilience*, with *ego resilience* having the lowest.

In response to the first research question, FL resilience is found to be a three-factor structure in the Chinese EFL learning contexts, including *ego resilience*, *metacognitive resilience* and *social resilience*, resonating Lazarus and Folkman (1984, p. 21) “personal, cognitive and situational appraisals” with regard to the theoretical assumptions of SCT. Like participants of the other contexts, FL learners who encounter diverse psychological issues and stressful events in FL learning are likely to trigger such personal attributes as perseverance, curiosity and energy (*ego resilience*), or seek for metacognitive strategies, such as goal setting, goal planning, help-seeking and control (*metacognitive resilience*), and establish positive social connections with other parties (*social resilience*). The three factors identified may inform pedagogy for EFL stakeholders on how to build resilient environments and develop learners’ resilience in times of FL learning adversities. A more informative interpretation of the three-factor structure could be achieved by comparing the structures with results of similar studies on FL resilience (Kim et al., 2019; Sudina and Plonsky, 2021). For instance, in a recent study, Sudina and Plonsky (2021) obtained two components of FL resilience: *coping with poor grades and criticism* and *dealing with study stress*, which is similar to *metacognitive resilience* of our study that highlights the use of metacognitive strategies to plan, monitor, evaluate and manage their FL learning difficulties or adversities (Li, 2022a). Likewise, to examine the impact of FL resilience on (de) motivation and language proficiency, Kim et al. (2019) directly used the domain-general academic resilience scale (Shin et al., 2009) to measure South Korean elementary school students’ FL resilience. The five-factor structure (*viz. metacognitive adaptation*, *sociability*, *optimism*, *perseverance*, and *communicative efficacy*) of their study could be further simplified as personal-related (*optimism* and *perseverance*), cognitive-related (*metacognitive adaptation* and *communicative efficacy*) and social related (*sociability*) resilience under the frame of Lazarus and Folkman (1984) SCT, suggesting that the three-factor structure of *FLLRS* in this study is more psychometrically elegant and simple.

The second question concerns the extent to which different factors predict the overall FL resilience. The CFA supports results obtained from the EFA, suggesting the psychometric validation of the 19-item *FLLRS* developed in this study. All the path coefficients of *metacognitive resilience* (*β* = 0.92, *p* < 0.001), *social resilience* (*β* = 0.83, *p* < 0.001) and *ego resilience* (*β* = 0.73, *p* < 0.001) have large effect sizes based on Plonsky and Oswald (2014) interpretations of the magnitude: 0.25, 0.40, and 0.60 for small, moderate, and large effects, respectively. However, when comparison of path coefficients was made among the three factors, *metacognitive resilience* was found to be the largest, followed by *social resilience*, with *ego resilience* having the lowest, indicating that EFL learners’ metacognitive resilience should be highlighted. In other words, to efficiently overcome adversities in FL learning, EFL learners should be sensitive to adopt various metacognitive strategies to plan, monitor, evaluate and manage their FL learning activities (Li, 2022a). Intriguingly, this result does not lend support to the descriptive statistic results with *ego resilience* (5.332 ± 1.083) being the largest, followed by *metacognitive resilience* (4.861 ± 1.234), and *social resilience* (4.748 ± 1.177) being the lowest. A plausible explanation for the discrepancy might be attributed to EFL learners themselves who intuitively tend to focus on personal appraisals first (Lazarus and Folkman, 1984), hence the highest self-report score of *ego resilience*. In other words, resilient EFL learners tend to first trust in their ability to recover from the adversities (Maltby et al., 2015), then begin to adopt positive cognitive strategies and confront with the challenging and stressful EFL learning situations (Cassidy, 2016).

## Practical implications, limitations and future directions

Some pedagogical implications could be inferred as follows. First, since the *FLLRS* has been validated, future study should examine the relationship between FL learners’ individual differences (e.g., age, gender, resilience levels and other demographic variables), FL contextual antecedents (e.g., learning environments, learning protocols and other contextual variables), and FL emotional factors in positive psychology (e.g., motivation, anxiety, boredom, enjoyment, well-being, engagement and flow experience, etc.) as has been done with academic resilience in the domain-general contexts. Second, the largest path coefficient of *metacognitive resilience* warrants the need to explore the predictive effects of *ego resilience*, *metacognitive resilience* and *social resilience* in general, and *metacognitive resilience* in particular on EFL learners’ learning aspects and FL performance. Such investigations are especially needed in contexts like China where FL learning is time-consuming with low efficiency (Li, 2021). To make positive adaptation to FL adversities, learners themselves should not only be perseverant in language learning, but also seek help from others and adopt some metacognitive strategies to monitor, evaluate and manage their FL learning behaviors and activities.

Despite the meaningful findings, limitations and future directions should be addressed though. First, this study only adopts a cross-sectional research design to understand the factors of FL resilience at one point in time, future research can adopt sophisticated research designs (e.g., longitudinal research design with mixed methods) to gain a better understanding of the diachronic changes of FL learners’ resilience over time. Second, this study is only based on samples of tertiary education level in the EFL learning contexts, its feasibility for primary and secondary educational level in other FL learning contexts remains open for future investigations. Third, while validity of the *FLLRS* is based on the homogenous data collected from Chinese EFL learners in Double First-Class universities, it remains largely unclear whether the *FLLRS* can be generalizable to other institutional contexts, e.g., non-Double First-Class or vocational universities, etc. Future studies should adopt a more comprehensive examination regarding the generalizability of the *FLLRS* across different levels of higher education institutions. Last and importantly, future research should adopt the state-of-the-art explanatory structural equation modeling (ESEM, see Alamer, 2022a,b for excellent methodological synergies) technique that combines both the EFA and CFA into one measurement model, which is a powerful technique in testing the construct validity of the second language acquisition scales in this regard.

## Data availability statement

The raw data supporting the conclusions of this article will be made available by the authors, without undue reservation.

## Ethics statement

The studies involving human participants were reviewed and approved by the ethics committee of Hunan University. The patients/participants provided their written informed consent to participate in this study.

## Author contributions

NG is responsible for the design, data collection, and drafting of the manuscript. RL is responsible for design and writing of the manuscript. All authors contributed to the article and approved the submitted version.

## Funding

This study was funded by the Project of Hunan Social Science Achievements Appraisal Committee (grant number XSP22YBC473) and the 14th Five Year Project for Education Science in Hunan Province (grant number XJK22CGD007).

## Conflict of interest

The authors declare that the research was conducted in the absence of any commercial or financial relationships that could be construed as a potential conflict of interest.

## Publisher’s note

All claims expressed in this article are solely those of the authors and do not necessarily represent those of their affiliated organizations, or those of the publisher, the editors and the reviewers. Any product that may be evaluated in this article, or claim that may be made by its manufacturer, is not guaranteed or endorsed by the publisher.

## Footnotes

^1^ https://www.wjx.cn/
