# Supplementary material for: Measuring Chinese English-as-a-foreign-language learners’ resilience: Development and validation of the foreign language learning resilience scale
Source: Front Psychol. 2022 Nov 28;13:1046340. doi: 10.3389/fpsyg.2022.1046340 (PMC9753691; doi:10.3389/fpsyg.2022.1046340)
Supplement: Supplementary file 1 [file Table_1.docx]

# Appendix 1

Initial items for the *FLLRS*

| **Factor** | **No.** | **Items in English** | **Items in Chinese** | **References** |
| --- | --- | --- | --- | --- |
| ER | 1 | I am curious about the new knowledge when I study a foreign language. | 当学一门外语时，我对学习外语新知识充满好奇。 | Block & Kreman (1996); Chen et al. (2020) |
|  | 2 | I am regarded as a very energetic person when I study a foreign language. | 当学一门外语时，我干劲十足。 |  |
|  | 3 | I am happy to make a favorable impression on others when I use a foreign language. | 当我因使用外语而给人留下好印象时，我倍感快乐。 |  |
|  | 4 (-) | I would be very disappointed when the scores I get in foreign language learning are low. | 当我外语成绩不理想时，我会倍感沮丧。 |  |
|  | 5 (-) | I am able to persevere even if it is not easy to learn a foreign language. | 即使学外语不那么容易，我也保持始终如一的认真态度。 |  |
|  | 6 | Perseverance matters, as it would never be easy to master a foreign language for a short period of time. | 我认为学外语不可一蹴而就，需要日积月累的坚持。 |  |
|  | 7 (-) | I would be resilient even though the scores I get in foreign language learning are low. | 即使外语成绩不理想，我也能很快从悲观中恢复过来。 |  |
|  | 8 (-) | I can cope with stress strengthens when the scores I get in foreign language learning are low. | 哪怕外语成绩不够理想，我也具有很强的抗压能力。 |  |
| MR | 9 | I would keep trying different methods to improve my foreign language. | 我会尝试各种不同的方法来提高外语能力。 | Conner & Davidson (2003); van de Meer et al. (2018); Wagnild & Young (1993) |
|  | 10 | I would use the feedback to improve my foreign language. | 我会利用反馈来提高外语能力。 |  |
|  | 11 | I would change my plans for language learning. | 为了提高外语能力，我会实时调整学习计划。 |  |
|  | 12 | I would learn from the excellent classmates to improve my foreign language. | 我会以学习好的同学为榜样，激励自己提高外语能力。 |  |
|  | 13 | I would try to think more about my strengths and weakness to help me study a foreign language better. | 我会分析自己的强项和弱点，以便更好地学习外语。 |  |
|  | 14 | I would set clear goals for achievements when I study a foreign language. | 为了提高外语能力，我会设定明确的目标。 |  |
|  | 15 | I am determined when I study a foreign language. | 当我决定学习一门外语时，我会想方设法坚持下去。 |  |
|  | 16 (-) | As for foreign language learning, I believe in myself gets me through hard times. | 就外语学习这件事而言，我坚信不经历风雨怎能见彩虹。 |  |
| SR | 17 | When I am encountered with difficulties in foreign language learning, I would seek encouragement from my classmates. | 当我在外语学习中遇到困难时，我会向同学寻求帮助。 | Cassidy (2016); Conner & Davidson (2003); Wagnild & Young (1993) |
|  | 18 | When I am encountered with difficulties in foreign language learning, I would seek help from my teachers. | 当我在外语学习中遇到困难时，我会向老师求助。 |  |
|  | 19 | When my classmate encountered with difficulties in foreign language learning, I would give a timely hand to help him/her. | 当别的同学在外语学习遇到问题时，我能及时给人力所能及的帮忙。 |  |
|  | 20 | Through foreign language learning, I can establish close and secure relationship with classmates. | 我与同学在学外语时互帮互助，相处融洽。 |  |
|  | 21 | It would be more efficient to ask others for help when I am encountered with difficulties in foreign language learning. | 我认为学外语有问题时询问别人会效率更高。 |  |
|  | 22 | It would be okay if there are people who do not like me, I still seek for help when I am encountered with difficulties in foreign language learning. | 我从来不管别人怎么看我，遇到不懂的我依然会请教别人。 |  |
|  | 23 | To well solve problems in foreign learning, I would try to make foreign friends if time permits. | 如果条件允许的话，我会尝试结交不少外国朋友，来解决外语学习问题。 |  |
|  | 24 | I would try collaborate with my classmates to complete foreign language learning tasks. | 我尽量跟同学合作来完成外语学习任务。 |  |

*Note*. ER = Ego resilience; MR = metacognitive resilience; SR = social resilience; (-) refers to the removal of item in the explanatory factor analysis.
